# A diastereomer of methyl (1*R*,3′*S*)-1′,1′′-dimethyl-2,2′′-dioxo-2*H*-dispiro­[ace­naphthyl­ene-1,2′-pyrrolidine-3′,3′′-indoline]-4′-carboxyl­ate

**DOI:** 10.1107/S1600536813004868

**Published:** 2013-02-23

**Authors:** Gnanavelu Ganesh, PanneerSelvam Yuvaraj, Piskala Subburaman Kannan, Boreddy Siva Rami Reddy, Arunachalathevar SubbiahPandi

**Affiliations:** a Department of Physics, S.M.K. Fomra Institute of Technology, Thaiyur, Chennai 603 103, India; b Industrial Chemistry Laboratory, Central Leather Research Institute, Adyar, Chennai 600 020, India; c Department of Physics, Presidency College (Autonomous), Chennai 600 005, India

## Abstract

In the title compound, C_26_H_22_N_2_O_4_, the central pyrrolidine ring adopts a twist conformation and the cyclo­pentane ring of the dihydro­acenapthylene group adopts an envelope conformation with the spiro C atom as the flap. The naphthalene ring system of the dihydro­acenaphthyl­ene group forms dihedral angles of 83.4 (9) and 61.3 (7)°, respectively, with the mean planes of the pyrrolidine and indole rings. The crystal packing is stabilized by inter­molecular C—H⋯O hydrogen bonds. The title compound is a diastereomer of a previously reported structure.

## Related literature

For background literature and the previously reported diastereomer, see: Ganesh *et al.* (2013 ▶). For a related structure, see: Wei *et al.* (2012 ▶). For information on ring conformations, see: Cremer & Pople (1975 ▶).
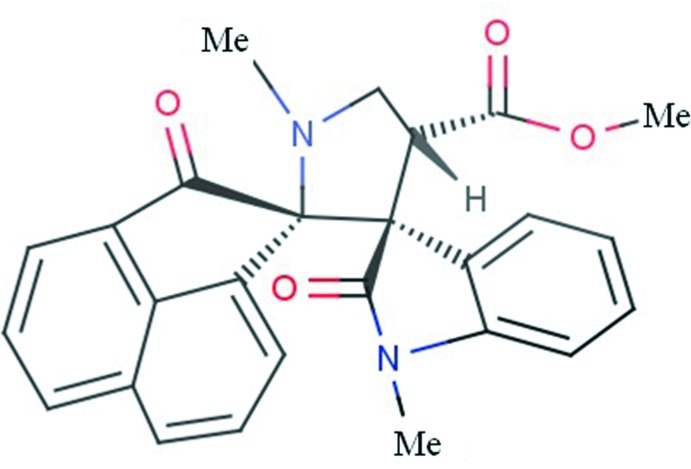


## Experimental

### 

#### Crystal data

C_26_H_22_N_2_O_4_
*M* *_r_* = 426.46
Orthorhombic, 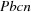

*a* = 27.2997 (15) Å
*b* = 9.7923 (6) Å
*c* = 15.8557 (10) Å
*V* = 4238.7 (4) Å^3^
*Z* = 8
Mo *K*α radiation
μ = 0.09 mm^−1^
*T* = 293 K
0.25 × 0.22 × 0.19 mm

#### Data collection

Bruker APEXII CCD area-detector diffractometer
Absorption correction: multi-scan (*SADABS*; Bruker, 2008 ▶) *T*_min_ = 0.978, *T*_max_ = 0.983
23813 measured reflections
5039 independent reflections
3022 reflections with *I* > 2σ(*I*)
*R*_int_ = 0.033

#### Refinement

*R*[*F* ^2^ > 2σ(*F* ^2^)] = 0.047
*wR*(*F* ^2^) = 0.135
*S* = 1.02
5039 reflections
292 parameters
H-atom parameters constrained
Δρ_max_ = 0.17 e Å^−3^
Δρ_min_ = −0.17 e Å^−3^

### 

Data collection: *APEX2* (Bruker, 2008 ▶); cell refinement: *SAINT*; data reduction: *SAINT*; program(s) used to solve structure: *SHELXS97* (Sheldrick, 2008 ▶); program(s) used to refine structure: *SHELXL97* (Sheldrick, 2008 ▶); molecular graphics: *ORTEP-3* (Farrugia, 2012 ▶); software used to prepare material for publication: *SHELXL97* and *PLATON* (Spek, 2009 ▶).

## Supplementary Material

Crystal structure: contains datablock(s) global, I. DOI: 10.1107/S1600536813004868/bt6885sup1.cif

Structure factors: contains datablock(s) I. DOI: 10.1107/S1600536813004868/bt6885Isup2.hkl

Additional supplementary materials:  crystallographic information; 3D view; checkCIF report

## Figures and Tables

**Table 1:** Hydrogen-bond geometry (Å, °)

*D*—H⋯*A*	*D*—H	H⋯*A*	*D*⋯*A*	*D*—H⋯*A*
C1—H1*B*⋯O3^i^	0.96	2.48	3.299 (3)	143
C20—H20⋯O3^ii^	0.93	2.59	3.403 (3)	146

Symmetry codes: (i) 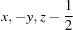; (ii) 
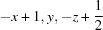
.

**Fig. 1.**
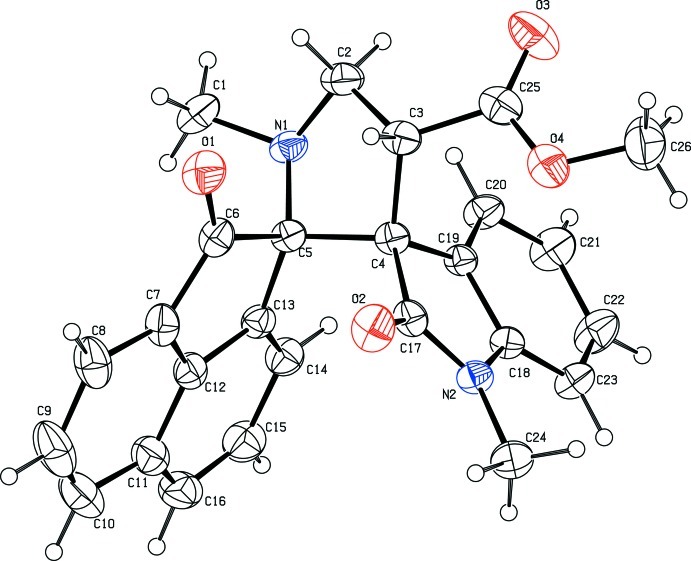
The molecular structure of the title compound with the atom numbering scheme. Displacement ellipsoids are drawn at the 30% probability level. H atoms are presented as small spheres of arbitrary radius.

**Fig. 2.**
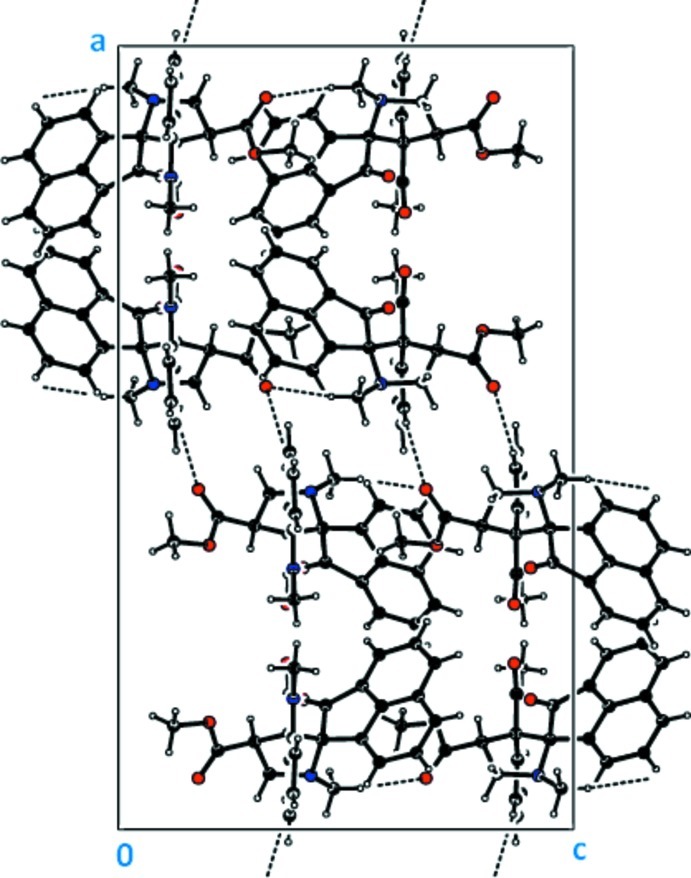
The molecular packing viewed down the b axis. Dashed lines shows the intermolecular C-H···O hydrogen bonds.

## supplementary crystallographic information

### Comment

We have recently reported the structure of a diastereomer of the title compound (Ganesh *et al.*, 2013). Here we report the structural details of its diastereomer.

X–Ray analysis confirms the molecular structure and atom connectivity as illustrated in Fig. 1. The geometry of the acenaphthylene and pyrrolidine ring systems are comparable with the related structure (Wei *et al.*, 2012). The sum of the angles at N1 [339.5 (1)°] and N2 [359.8 (1)°] of the pyrrolidine rings are in accordance with *sp*^3^ and *sp*^2^ hybridization. The naphthalene ring system [C7–C16] of the dihydroacenaphthylene group forms dihedral angles of 83.4 (9) and 61.3 (7)° with the central pyrrolidine ring [N1/C2–C5] and the indole ring [N2/C4/C17–C23]. The central pyrrolidine also makes a dihedral angle of 87.6 (9)° with the indole which shows that these two rings are almost perpendicular with each other.

The pyrrolidine rings [N1/C2-C5] adopts a twist conformation, on C4 and C5 atoms with puckering parameters of q_2_ = 0.470 (2) Å, φ = 128.1 (2) (Cremer & Pople, 1975). The cyclopentane ring [C5-C7/C12-C13] in the dihydroacenapthylene group adopts an envelope conformation [q_2_ = 0.074 (9)(2) Å and φ = 173.3 (2)°], and with atom C5 deviating by 0.044 (2) Å from the least-squares plane passing through the remaining four atoms (C13/C12/C7/C6) of that ring. In the crystal, the molecules are linked by intermolecular C20-H20···O3 and C1-H1B···O3 hydrogen bonds Fig. 2.

### Experimental

A mixture of 1 eq of (*E*)-methyl 2-(1-methyl-2-oxoindolin-3-ylidene) acetate, 1 eq of isatin and 1.5 eq of acenaphthylene-1,2-dione were dissolved in acetonitrile. This reaction mixture was refluxed at 353K for 8 hours. The reaction mixture was monitored for completion by thin layer chromatography. Upon completion, the the product was dried and purified by coloumn chromatography using ethyl acetate and hexane (1:9) as an elutent to affored pure dispiro oxindole. Yield (78%). Single crystals suitable for X-ray diffraction were obtained by slow evaporation of a solution of the title compound in ethyl acetate at room temperature.

### Refinement

All H atoms were fixed geometrically and allowed to ride on their parent C atoms, with C—H distances fixed in the range 0.93–0.98 Å with *U*_iso_(H) = 1.5*U*_eq_(C) for methyl H 1.2*U*_eq_(C) for other H atoms.

### Crystal data

**Table:**

C_26_H_22_N_2_O_4_	*F*(000) = 1792
*M**_r_* = 426.46	*D*_x_ = 1.337 Mg m^−^^3^
Orthorhombic, *P**b**c**n*	Mo *K*α radiation, λ = 0.71073 Å
Hall symbol: -P 2n 2ab	Cell parameters from 5039 reflections
*a* = 27.2997 (15) Å	θ = 1.5–27.9°
*b* = 9.7923 (6) Å	µ = 0.09 mm^−^^1^
*c* = 15.8557 (10) Å	*T* = 293 K
*V* = 4238.7 (4) Å^3^	Block, colourless
*Z* = 8	0.25 × 0.22 × 0.19 mm

### Data collection

**Table:**

Bruker APEXII CCD area-detector diffractometer	5039 independent reflections
Radiation source: fine-focus sealed tube	3022 reflections with *I* > 2σ(*I*)
Graphite monochromator	*R*_int_ = 0.033
ω and φ scans	θ_max_ = 27.9°, θ_min_ = 2.2°
Absorption correction: multi-scan (*SADABS*; Bruker, 2008)	*h* = −35→31
*T*_min_ = 0.978, *T*_max_ = 0.983	*k* = −12→11
23813 measured reflections	*l* = −18→20

### Refinement

**Table:**

Refinement on *F*^2^	Primary atom site location: structure-invariant direct methods
Least-squares matrix: full	Secondary atom site location: difference Fourier map
*R*[*F*^2^ > 2σ(*F*^2^)] = 0.047	Hydrogen site location: inferred from neighbouring sites
*wR*(*F*^2^) = 0.135	H-atom parameters constrained
*S* = 1.02	*w* = 1/[σ^2^(*F*_o_^2^) + (0.0531*P*)^2^ + 1.3523*P*] where *P* = (*F*_o_^2^ + 2*F*_c_^2^)/3
5039 reflections	(Δ/σ)_max_ = 0.002
292 parameters	Δρ_max_ = 0.17 e Å^−^^3^
0 restraints	Δρ_min_ = −0.17 e Å^−^^3^

### Special details

**Table:**

Geometry. All esds (except the esd in the dihedral angle between two l.s. planes) are estimated using the full covariance matrix. The cell esds are taken into account individually in the estimation of esds in distances, angles and torsion angles; correlations between esds in cell parameters are only used when they are defined by crystal symmetry. An approximate (isotropic) treatment of cell esds is used for estimating esds involving l.s. planes.
Refinement. Refinement of F^2^ against ALL reflections. The weighted R-factor wR and goodness of fit S are based on F^2^, conventional R-factors R are based on F, with F set to zero for negative F^2^. The threshold expression of F^2^ > 2sigma(F^2^) is used only for calculating R-factors(gt) etc. and is not relevant to the choice of reflections for refinement. R-factors based on F^2^ are statistically about twice as large as those based on F, and R- factors based on ALL data will be even larger.

### Fractional atomic coordinates and isotropic or equivalent isotropic displacement parameters (Å^2^)

**Table:**

	*x*	*y*	*z*	*U*_iso_*/*U*_eq_	
C1	0.55248 (7)	−0.08437 (18)	0.02663 (15)	0.0583 (6)	
H1A	0.5740	−0.1607	0.0354	0.087*	
H1B	0.5527	−0.0596	−0.0320	0.087*	
H1C	0.5198	−0.1088	0.0432	0.087*	
C2	0.57113 (7)	0.0054 (2)	0.16787 (14)	0.0543 (5)	
H2A	0.5784	−0.0897	0.1794	0.065*	
H2B	0.5402	0.0285	0.1943	0.065*	
C3	0.61210 (7)	0.09760 (19)	0.20075 (12)	0.0463 (5)	
H3	0.6412	0.0409	0.2098	0.056*	
C4	0.62250 (6)	0.19357 (17)	0.12618 (11)	0.0380 (4)	
C5	0.61516 (6)	0.09285 (16)	0.05178 (12)	0.0380 (4)	
C6	0.65873 (6)	−0.01367 (18)	0.04464 (13)	0.0454 (5)	
C7	0.68374 (6)	0.0100 (2)	−0.03587 (13)	0.0492 (5)	
C8	0.72342 (7)	−0.0500 (2)	−0.07337 (16)	0.0672 (6)	
H8	0.7404	−0.1202	−0.0469	0.081*	
C9	0.73756 (9)	−0.0022 (3)	−0.15287 (18)	0.0842 (8)	
H9	0.7652	−0.0399	−0.1781	0.101*	
C10	0.71257 (9)	0.0973 (3)	−0.19492 (16)	0.0778 (7)	
H10	0.7234	0.1258	−0.2477	0.093*	
C11	0.67050 (8)	0.1574 (2)	−0.15918 (14)	0.0573 (5)	
C12	0.65758 (7)	0.11197 (19)	−0.07840 (12)	0.0444 (4)	
C13	0.61607 (6)	0.15884 (17)	−0.03457 (12)	0.0401 (4)	
C14	0.58614 (7)	0.24994 (19)	−0.07357 (13)	0.0500 (5)	
H14	0.5581	0.2818	−0.0467	0.060*	
C15	0.59812 (9)	0.2955 (2)	−0.15527 (14)	0.0617 (6)	
H15	0.5773	0.3571	−0.1819	0.074*	
C16	0.63878 (9)	0.2529 (2)	−0.19651 (14)	0.0661 (6)	
H16	0.6458	0.2872	−0.2499	0.079*	
C17	0.67325 (6)	0.26047 (19)	0.12476 (12)	0.0413 (4)	
C18	0.61710 (6)	0.43187 (17)	0.11591 (11)	0.0368 (4)	
C19	0.58897 (6)	0.31498 (16)	0.12240 (11)	0.0359 (4)	
C20	0.53874 (6)	0.32524 (19)	0.12696 (13)	0.0479 (5)	
H20	0.5193	0.2477	0.1322	0.058*	
C21	0.51788 (7)	0.4538 (2)	0.12360 (16)	0.0627 (6)	
H21	0.4840	0.4627	0.1268	0.075*	
C22	0.54625 (8)	0.5680 (2)	0.11568 (16)	0.0644 (6)	
H22	0.5313	0.6532	0.1132	0.077*	
C23	0.59676 (7)	0.55977 (19)	0.11128 (13)	0.0501 (5)	
H23	0.6161	0.6374	0.1054	0.060*	
C24	0.70630 (7)	0.4956 (2)	0.11344 (14)	0.0560 (5)	
H24A	0.7370	0.4487	0.1084	0.084*	
H24B	0.7060	0.5481	0.1646	0.084*	
H24C	0.7020	0.5554	0.0660	0.084*	
C25	0.60097 (8)	0.1700 (2)	0.28144 (14)	0.0548 (5)	
C26	0.63246 (11)	0.3314 (3)	0.37857 (16)	0.0865 (8)	
H26A	0.5988	0.3558	0.3876	0.130*	
H26B	0.6520	0.4127	0.3756	0.130*	
H26C	0.6436	0.2755	0.4245	0.130*	
N1	0.56904 (5)	0.03024 (14)	0.07673 (10)	0.0434 (4)	
N2	0.66700 (5)	0.39740 (15)	0.11545 (9)	0.0403 (4)	
O1	0.66576 (5)	−0.10603 (14)	0.09387 (10)	0.0617 (4)	
O2	0.71209 (5)	0.20188 (14)	0.12955 (10)	0.0625 (4)	
O3	0.56571 (6)	0.1529 (2)	0.32417 (11)	0.0878 (6)	
O4	0.63683 (6)	0.25647 (16)	0.30058 (10)	0.0693 (4)	

### Atomic displacement parameters (Å^2^)

**Table:**

	*U*^11^	*U*^22^	*U*^33^	*U*^12^	*U*^13^	*U*^23^
C1	0.0490 (11)	0.0381 (10)	0.0879 (17)	−0.0046 (9)	−0.0040 (11)	0.0022 (10)
C2	0.0531 (12)	0.0480 (11)	0.0617 (14)	−0.0003 (10)	0.0105 (10)	0.0145 (10)
C3	0.0414 (10)	0.0462 (10)	0.0512 (12)	0.0086 (8)	0.0057 (9)	0.0108 (9)
C4	0.0278 (8)	0.0392 (9)	0.0470 (11)	0.0050 (7)	0.0022 (7)	0.0035 (8)
C5	0.0293 (8)	0.0356 (8)	0.0491 (11)	0.0030 (7)	0.0016 (8)	0.0035 (8)
C6	0.0369 (9)	0.0405 (10)	0.0588 (12)	0.0054 (8)	−0.0038 (9)	−0.0033 (9)
C7	0.0354 (9)	0.0547 (11)	0.0576 (13)	0.0022 (9)	−0.0015 (9)	−0.0152 (10)
C8	0.0456 (12)	0.0851 (16)	0.0708 (16)	0.0130 (11)	0.0014 (11)	−0.0249 (13)
C9	0.0501 (14)	0.130 (2)	0.0731 (18)	0.0053 (16)	0.0132 (13)	−0.0356 (18)
C10	0.0615 (15)	0.118 (2)	0.0535 (15)	−0.0135 (15)	0.0146 (12)	−0.0166 (15)
C11	0.0541 (12)	0.0691 (14)	0.0487 (13)	−0.0160 (11)	0.0033 (10)	−0.0122 (11)
C12	0.0383 (10)	0.0490 (10)	0.0460 (11)	−0.0077 (8)	−0.0008 (8)	−0.0098 (9)
C13	0.0346 (9)	0.0391 (9)	0.0465 (11)	−0.0042 (7)	−0.0007 (8)	−0.0018 (8)
C14	0.0522 (11)	0.0468 (10)	0.0510 (12)	0.0023 (9)	−0.0042 (9)	0.0033 (9)
C15	0.0739 (15)	0.0565 (12)	0.0546 (14)	−0.0030 (11)	−0.0114 (12)	0.0076 (10)
C16	0.0824 (16)	0.0717 (15)	0.0443 (13)	−0.0195 (14)	−0.0002 (12)	0.0046 (11)
C17	0.0298 (9)	0.0503 (10)	0.0439 (11)	0.0042 (8)	0.0005 (8)	−0.0039 (8)
C18	0.0312 (9)	0.0406 (9)	0.0388 (10)	0.0001 (7)	−0.0009 (7)	0.0022 (7)
C19	0.0302 (8)	0.0364 (9)	0.0410 (10)	0.0028 (7)	0.0035 (7)	0.0027 (7)
C20	0.0287 (9)	0.0435 (10)	0.0716 (14)	0.0002 (8)	0.0057 (9)	0.0043 (9)
C21	0.0327 (10)	0.0522 (12)	0.1033 (19)	0.0103 (9)	0.0043 (11)	0.0031 (12)
C22	0.0507 (12)	0.0411 (11)	0.1013 (19)	0.0123 (10)	−0.0040 (12)	0.0057 (11)
C23	0.0472 (11)	0.0385 (10)	0.0646 (14)	−0.0027 (8)	−0.0034 (10)	0.0049 (9)
C24	0.0359 (10)	0.0637 (12)	0.0684 (15)	−0.0142 (9)	0.0005 (10)	−0.0036 (11)
C25	0.0512 (12)	0.0612 (13)	0.0522 (13)	0.0139 (10)	0.0039 (10)	0.0137 (10)
C26	0.113 (2)	0.0783 (17)	0.0680 (17)	0.0095 (16)	0.0000 (16)	−0.0145 (14)
N1	0.0343 (8)	0.0364 (8)	0.0595 (10)	−0.0005 (6)	0.0051 (7)	0.0061 (7)
N2	0.0283 (7)	0.0451 (8)	0.0474 (9)	−0.0041 (6)	−0.0004 (6)	0.0007 (7)
O1	0.0559 (9)	0.0494 (8)	0.0797 (11)	0.0188 (7)	0.0000 (8)	0.0083 (8)
O2	0.0301 (7)	0.0664 (9)	0.0908 (12)	0.0120 (6)	−0.0030 (7)	−0.0080 (8)
O3	0.0700 (11)	0.1265 (15)	0.0668 (11)	0.0017 (10)	0.0238 (9)	−0.0047 (11)
O4	0.0766 (11)	0.0749 (10)	0.0566 (10)	−0.0028 (9)	0.0069 (8)	−0.0051 (8)

### Geometric parameters (Å, º)

**Table:**

C1—N1	1.447 (2)	C13—C14	1.359 (2)
C1—H1A	0.9600	C14—C15	1.408 (3)
C1—H1B	0.9600	C14—H14	0.9300
C1—H1C	0.9600	C15—C16	1.354 (3)
C2—N1	1.467 (3)	C15—H15	0.9300
C2—C3	1.529 (3)	C16—H16	0.9300
C2—H2A	0.9700	C17—O2	1.208 (2)
C2—H2B	0.9700	C17—N2	1.360 (2)
C3—C25	1.494 (3)	C18—C23	1.372 (2)
C3—C4	1.537 (2)	C18—C19	1.382 (2)
C3—H3	0.9800	C18—N2	1.403 (2)
C4—C19	1.502 (2)	C19—C20	1.377 (2)
C4—C17	1.533 (2)	C20—C21	1.383 (3)
C4—C5	1.551 (2)	C20—H20	0.9300
C5—N1	1.455 (2)	C21—C22	1.366 (3)
C5—C13	1.514 (2)	C21—H21	0.9300
C5—C6	1.586 (2)	C22—C23	1.383 (3)
C6—O1	1.210 (2)	C22—H22	0.9300
C6—C7	1.466 (3)	C23—H23	0.9300
C7—C8	1.368 (3)	C24—N2	1.441 (2)
C7—C12	1.401 (3)	C24—H24A	0.9600
C8—C9	1.399 (4)	C24—H24B	0.9600
C8—H8	0.9300	C24—H24C	0.9600
C9—C10	1.364 (4)	C25—O3	1.189 (2)
C9—H9	0.9300	C25—O4	1.330 (3)
C10—C11	1.410 (3)	C26—O4	1.443 (3)
C10—H10	0.9300	C26—H26A	0.9600
C11—C12	1.401 (3)	C26—H26B	0.9600
C11—C16	1.406 (3)	C26—H26C	0.9600
C12—C13	1.406 (2)		
			
N1—C1—H1A	109.5	C12—C13—C5	108.70 (15)
N1—C1—H1B	109.5	C13—C14—C15	119.1 (2)
H1A—C1—H1B	109.5	C13—C14—H14	120.4
N1—C1—H1C	109.5	C15—C14—H14	120.4
H1A—C1—H1C	109.5	C16—C15—C14	122.5 (2)
H1B—C1—H1C	109.5	C16—C15—H15	118.8
N1—C2—C3	105.47 (15)	C14—C15—H15	118.8
N1—C2—H2A	110.6	C15—C16—C11	120.4 (2)
C3—C2—H2A	110.6	C15—C16—H16	119.8
N1—C2—H2B	110.6	C11—C16—H16	119.8
C3—C2—H2B	110.6	O2—C17—N2	125.81 (17)
H2A—C2—H2B	108.8	O2—C17—C4	126.13 (17)
C25—C3—C2	115.06 (17)	N2—C17—C4	108.05 (14)
C25—C3—C4	113.98 (16)	C23—C18—C19	122.33 (16)
C2—C3—C4	103.53 (15)	C23—C18—N2	127.75 (16)
C25—C3—H3	108.0	C19—C18—N2	109.91 (14)
C2—C3—H3	108.0	C20—C19—C18	119.80 (15)
C4—C3—H3	108.0	C20—C19—C4	131.52 (15)
C19—C4—C17	102.25 (13)	C18—C19—C4	108.64 (14)
C19—C4—C3	113.70 (14)	C19—C20—C21	118.33 (17)
C17—C4—C3	116.09 (15)	C19—C20—H20	120.8
C19—C4—C5	113.23 (14)	C21—C20—H20	120.8
C17—C4—C5	112.19 (14)	C22—C21—C20	121.03 (18)
C3—C4—C5	99.93 (13)	C22—C21—H21	119.5
N1—C5—C13	116.08 (14)	C20—C21—H21	119.5
N1—C5—C4	99.96 (13)	C21—C22—C23	121.47 (18)
C13—C5—C4	114.48 (13)	C21—C22—H22	119.3
N1—C5—C6	113.03 (13)	C23—C22—H22	119.3
C13—C5—C6	101.76 (14)	C18—C23—C22	117.02 (18)
C4—C5—C6	112.06 (14)	C18—C23—H23	121.5
O1—C6—C7	127.30 (17)	C22—C23—H23	121.5
O1—C6—C5	124.36 (17)	N2—C24—H24A	109.5
C7—C6—C5	107.91 (15)	N2—C24—H24B	109.5
C8—C7—C12	120.0 (2)	H24A—C24—H24B	109.5
C8—C7—C6	132.8 (2)	N2—C24—H24C	109.5
C12—C7—C6	107.11 (16)	H24A—C24—H24C	109.5
C7—C8—C9	117.8 (2)	H24B—C24—H24C	109.5
C7—C8—H8	121.1	O3—C25—O4	123.7 (2)
C9—C8—H8	121.1	O3—C25—C3	125.9 (2)
C10—C9—C8	122.8 (2)	O4—C25—C3	110.38 (17)
C10—C9—H9	118.6	O4—C26—H26A	109.5
C8—C9—H9	118.6	O4—C26—H26B	109.5
C9—C10—C11	120.6 (2)	H26A—C26—H26B	109.5
C9—C10—H10	119.7	O4—C26—H26C	109.5
C11—C10—H10	119.7	H26A—C26—H26C	109.5
C12—C11—C16	116.18 (19)	H26B—C26—H26C	109.5
C12—C11—C10	116.1 (2)	C1—N1—C5	116.59 (15)
C16—C11—C10	127.6 (2)	C1—N1—C2	115.12 (15)
C7—C12—C11	122.55 (18)	C5—N1—C2	107.69 (14)
C7—C12—C13	113.93 (18)	C17—N2—C18	111.00 (14)
C11—C12—C13	123.44 (19)	C17—N2—C24	124.54 (15)
C14—C13—C12	118.29 (18)	C18—N2—C24	124.22 (15)
C14—C13—C5	133.00 (17)	C25—O4—C26	117.31 (19)
			
N1—C2—C3—C25	137.57 (16)	C12—C13—C14—C15	−1.0 (3)
N1—C2—C3—C4	12.55 (18)	C5—C13—C14—C15	178.08 (18)
C25—C3—C4—C19	−41.3 (2)	C13—C14—C15—C16	−0.7 (3)
C2—C3—C4—C19	84.45 (18)	C14—C15—C16—C11	1.5 (3)
C25—C3—C4—C17	77.0 (2)	C12—C11—C16—C15	−0.6 (3)
C2—C3—C4—C17	−157.33 (15)	C10—C11—C16—C15	176.3 (2)
C25—C3—C4—C5	−162.21 (15)	C19—C4—C17—O2	177.45 (19)
C2—C3—C4—C5	−36.50 (16)	C3—C4—C17—O2	53.1 (3)
C19—C4—C5—N1	−73.87 (16)	C5—C4—C17—O2	−60.9 (2)
C17—C4—C5—N1	171.02 (13)	C19—C4—C17—N2	−3.77 (19)
C3—C4—C5—N1	47.41 (14)	C3—C4—C17—N2	−128.12 (16)
C19—C4—C5—C13	50.91 (19)	C5—C4—C17—N2	117.86 (16)
C17—C4—C5—C13	−64.20 (18)	C23—C18—C19—C20	−2.0 (3)
C3—C4—C5—C13	172.20 (14)	N2—C18—C19—C20	177.74 (17)
C19—C4—C5—C6	166.14 (14)	C23—C18—C19—C4	−179.96 (17)
C17—C4—C5—C6	51.03 (19)	N2—C18—C19—C4	−0.3 (2)
C3—C4—C5—C6	−72.58 (16)	C17—C4—C19—C20	−175.3 (2)
N1—C5—C6—O1	−40.9 (3)	C3—C4—C19—C20	−49.4 (3)
C13—C5—C6—O1	−166.14 (18)	C5—C4—C19—C20	63.8 (3)
C4—C5—C6—O1	71.1 (2)	C17—C4—C19—C18	2.39 (18)
N1—C5—C6—C7	131.98 (16)	C3—C4—C19—C18	128.31 (16)
C13—C5—C6—C7	6.77 (17)	C5—C4—C19—C18	−118.52 (16)
C4—C5—C6—C7	−115.98 (16)	C18—C19—C20—C21	0.9 (3)
O1—C6—C7—C8	−8.3 (4)	C4—C19—C20—C21	178.4 (2)
C5—C6—C7—C8	179.0 (2)	C19—C20—C21—C22	0.2 (3)
O1—C6—C7—C12	168.86 (19)	C20—C21—C22—C23	−0.4 (4)
C5—C6—C7—C12	−3.79 (19)	C19—C18—C23—C22	1.7 (3)
C12—C7—C8—C9	2.4 (3)	N2—C18—C23—C22	−177.93 (19)
C6—C7—C8—C9	179.3 (2)	C21—C22—C23—C18	−0.5 (3)
C7—C8—C9—C10	−2.3 (4)	C2—C3—C25—O3	6.7 (3)
C8—C9—C10—C11	0.0 (4)	C4—C3—C25—O3	126.1 (2)
C9—C10—C11—C12	2.0 (3)	C2—C3—C25—O4	−174.36 (16)
C9—C10—C11—C16	−174.9 (2)	C4—C3—C25—O4	−55.0 (2)
C8—C7—C12—C11	−0.4 (3)	C13—C5—N1—C1	63.6 (2)
C6—C7—C12—C11	−178.03 (17)	C4—C5—N1—C1	−172.74 (14)
C8—C7—C12—C13	176.48 (17)	C6—C5—N1—C1	−53.5 (2)
C6—C7—C12—C13	−1.1 (2)	C13—C5—N1—C2	−165.25 (15)
C16—C11—C12—C7	175.42 (18)	C4—C5—N1—C2	−41.59 (16)
C10—C11—C12—C7	−1.8 (3)	C6—C5—N1—C2	77.70 (18)
C16—C11—C12—C13	−1.2 (3)	C3—C2—N1—C1	150.60 (15)
C10—C11—C12—C13	−178.42 (18)	C3—C2—N1—C5	18.64 (18)
C7—C12—C13—C14	−174.87 (16)	O2—C17—N2—C18	−177.33 (18)
C11—C12—C13—C14	2.0 (3)	C4—C17—N2—C18	3.9 (2)
C7—C12—C13—C5	5.8 (2)	O2—C17—N2—C24	−2.8 (3)
C11—C12—C13—C5	−177.31 (16)	C4—C17—N2—C24	178.40 (16)
N1—C5—C13—C14	50.3 (3)	C23—C18—N2—C17	177.32 (19)
C4—C5—C13—C14	−65.5 (2)	C19—C18—N2—C17	−2.4 (2)
C6—C5—C13—C14	173.43 (19)	C23—C18—N2—C24	2.8 (3)
N1—C5—C13—C12	−130.55 (15)	C19—C18—N2—C24	−176.90 (17)
C4—C5—C13—C12	113.69 (15)	O3—C25—O4—C26	1.0 (3)
C6—C5—C13—C12	−7.40 (17)	C3—C25—O4—C26	−177.94 (18)

### Hydrogen-bond geometry (Å, º)

**Table:**

*D*—H···*A*	*D*—H	H···*A*	*D*···*A*	*D*—H···*A*
C1—H1*B*···O3^i^	0.96	2.48	3.299 (3)	143
C20—H20···O3^ii^	0.93	2.59	3.403 (3)	146

Symmetry codes: (i) *x*, −*y*, *z*−1/2; (ii) −*x*+1, *y*, −*z*+1/2.
